# Dual Element (C/Cl) Isotope Analysis Indicates Distinct Mechanisms of Reductive Dehalogenation of Chlorinated Ethenes and Dichloroethane in *Dehalococcoides mccartyi* Strain BTF08 With Defined Reductive Dehalogenase Inventories

**DOI:** 10.3389/fmicb.2020.01507

**Published:** 2020-07-17

**Authors:** Steffi Franke, Katja Seidel, Lorenz Adrian, Ivonne Nijenhuis

**Affiliations:** ^1^Department of Isotope Biogeochemistry, Helmholtz Centre for Environmental Research – UFZ, Leipzig, Germany; ^2^Chair of Geobiotechnology at TU Berlin, Berlin, Germany

**Keywords:** compound-specific stable isotope analysis, nLC-MS/MS, *Dehalococcoides mccartyi* strain BTF08, reductive dehalogenation, 1, 2-dichloroethane, tetrachloroethene, *cis*-dichloroethene, vinyl chloride

## Abstract

*Dehalococcoides mccartyi* strain BTF08 has the unique property to couple complete dechlorination of tetrachloroethene and 1,2-dichloroethane to ethene with growth by using the halogenated compounds as terminal electron acceptor. The genome of strain BTF08 encodes 20 genes for reductive dehalogenase homologous proteins (RdhA) including those described for dehalogenation of tetrachloroethene (PceA, PteA), trichloroethene (TceA) and vinyl chloride (VcrA). Thus far it is unknown under which conditions the different RdhAs are expressed, what their substrate specificity is and if different reaction mechanisms are employed. Here we found by proteomic analysis from differentially activated batches that PteA and VcrA were expressed during dechlorination of tetrachloroethene to ethene, while TceA was expressed during 1,2-dichloroethane dehalogenation. Carbon and chlorine compound-specific stable isotope analysis suggested distinct reaction mechanisms for the dechlorination of (i) *cis*-dichloroethene and vinyl chloride versus (ii) tetrachloroethene. This differentiation was observed independent of the expressed RdhA proteins. Differently, two stable isotope fractionation patterns were observed for 1,2-dichloroethane transformation, for cells with distinct RdhA inventories. Conclusively, we could link specific RdhA expression with functions and provide an insight into the apparently substrate-specific reaction mechanisms in the pathway of reductive dehalogenation in *D. mccartyi* strain BTF08. Data are available via ProteomeXchange with identifiers PXD018558 and PXD018595.

## Introduction

Chlorinated organic contaminants, such as tetrachloroethene (PCE), belong to the most frequent groundwater contaminants entering the environment through accidents, improper handling and waste disposal (Carter et al., 2008). Thus far, only microorganisms belonging to the class *Dehalococcoidia* can catalyze complete dehalogenation of PCE to the non-toxic ethene and have been shown to be key players for *in situ* bioremediation of chlorinated solvents (Löffler et al., 2013). *Dehalococcoides mccartyi* strain BTF08 harbors the unique property to couple all reductive dehalogenation steps starting from PCE down to ethene to growth referred to as organohalide respiration (Cichocka et al., 2010; Kaufhold et al., 2013). Further, strain BTF08 is capable of dehalogenating vicinal halogenated alkanes to ethene (Schmidt et al., 2014). Its genome contains 20 reductive dehalogenase homologous gene clusters (*rdhAB*) wherefrom 16 *rdhAB* genes belong to classified orthologue groups being conserved throughout cultivated *D. mccartyi* strains (Hug et al., 2013). The *rdhAB* genes in strain BTF08 include orthologues of *pceA, tceA*, *and vcrA*, proposed to be involved in PCE, TCE, and vinyl chloride (VC) dehalogenation, respectively, while the other *rdhA* genes can currently not be functionally assigned. Thus, *D. mccartyi* strain BTF08 possesses all RdhA encoding genes required for complete reductive dehalogenation of PCE to ethene (Pöritz et al., 2013). Functional annotation of the *rdhA* genes in strain BTF08 has been done on the basis of their affiliation to orthologue groups containing biochemically characterized orthologues in other *D. mccartyi* strains (Hug et al., 2013), but the biochemical activity has not been confirmed experimentally in *D. mccartyi* strain BTF08.

**FIGURE 1:**
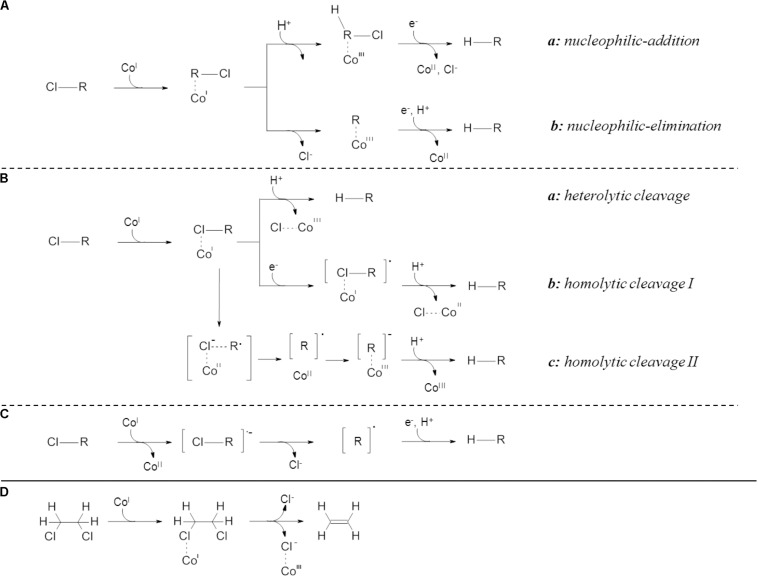
Proposed mechanisms for reductive dehalogenation. **(A)** inner sphere two electron transfers, **(B)** inner sphere single electron transfers displaying different modes of bond cleavage, **(C)** outer sphere single electron transfer and **(D)** inner sphere single electron transfer for vicinally substituted compounds. Inner sphere two electron transfer **(A)** is further divided in nucleophilic-addition reaction **(a)**, characterized by initial protonation followed by carbon-halogen bond cleavage, and nucleophilic elimination **(b)**, possessing initial carbon-halogen bond cleavage with subsequent protonation of the remaining carbon (Heckel et al., 2018; Lihl et al., 2019). Inner sphere single electron transfers **(B)** are described as heterolytic **(a)** or homolytic **(b,c)** cleavage (Cooper et al., 2015; Payne et al., 2015). Two distinct mechanisms of homolytic cleavage are proposed leading either to abstraction of a cobalt-halogen complex (Cooper et al., 2015; Payne et al., 2015) **(b)** or association of the carbon backbone to the cobalt (Cooper et al., 2015; Payne et al., 2015) **(c)**. Outer sphere single electron transfers **(C)** do not involve a carbon or halogen to cobalt interaction (Kunze et al., 2017). Inner sphere single electron transfer for vicinally substituted compounds **(D)** was proposed for concerted halogen removal (Payne et al., 2015). Displayed are the hydrocarbon backbone [R] and the oxidation states of the cobalt within the cobamid cofactor [Co^*I–III*^].

Among all RdhA several conserved features were identified, including a twin-arginine signal motif for cytoplasmic membrane translocation, two iron-sulfur cluster binding motifs as well as a motif for a corrinoid-containing co-factor (Magnuson et al., 2000; Holscher et al., 2004). Based on investigation with the corrinoid cyanocobalamin (Vitamin B_12_), a super-reduced Co(I) species was shown to be involved in the catalytic activity of RdhA (Miller et al., 1996; Schumacher et al., 1997; Middeldorp et al., 1999; Parthasarathy et al., 2015; Kunze et al., 2017). Reactions with the pure corrinoid were proposed to take place as (i) inner sphere single electron transfer (SET) (Cooper et al., 2015; Payne et al., 2015), (ii) inner sphere two electron transfer (Kliegman and McNeill, 2008) or (iii) long range single electron transfer (Bommer et al., 2014; Figure 1). Studies on structurally characterized RdhA proposed either long range SET for PceA from *Sulfurospirillum multivorans* (Bommer et al., 2014) and VcrA from *Dehalococcoides mccartyi* strain VS (Parthasarathy et al., 2015) or inner sphere SET for NpRdhA from *Nitratireductor pacificus* (Payne et al., 2015). Inner sphere two electron transfer was recently proposed in studies investigating biotic and abiotic dehalogenation of halogenated ethenes using compound-specific stable isotope analysis (CSIA) (Heckel et al., 2017, 2018; Lihl et al., 2019).

Compound-specific stable isotope analysis emerged as powerful tool to evaluate reaction mechanisms (Elsner et al., 2005; Hirschorn et al., 2007; Schmidt et al., 2014), allowing to relate isotope fractionation patterns with distinct reactions and characterization of the rate-determining step (Vogt et al., 2008). Recently, the absence of chlorine isotope fractionation was shown for outer-sphere SET on halogenated ethenes (Heckel et al., 2017). Additionally, a dichotomy in the reaction mechanisms during TCE-dehalogenation was observed in dependence on the electron acceptor used in the previous cultivation for several *D. mccartyi* containing mixed cultures and *Geobacter lovleyi* strain KB-1 (Lihl et al., 2019). The involved electron transfer reactions were proposed to be inner sphere two electron transfers (Figure 1A). However, this was not linked to specific microorganisms or dehalogenases (Lihl et al., 2019). Thus, we hypothesized the presence of distinct, electron-acceptor specific reaction mechanisms during corrinoid-dependent dechlorination at their respective RdhAs.

Here we aimed to characterize the reaction mechanisms during dehalogenation of PCE, cDCE, VC, and 1,2-dichloroethane (1,2-DCA) using dual element (C/Cl) CSIA. Furthermore we aimed to investigate the RdhA inventory present in the underlying dehalogenation processes by experiments.

## Materials and Methods

### Cultivation of *Dehalococcoides mccartyi* Strain BTF08

*Dehalococcoides mccartyi* strain BTF08 was pre-cultivated for at least 4 transfers in 100 mL mineral medium (final pH 6.8) in 240 mL serum bottles and in total 1.2 mM PCE, *cis*-DCE, 1,2-DCA or VC as electron acceptor prior to transfer 0 (Supplementary Figure S1A) as described elsewhere (Schmidt et al., 2014; Franke et al., 2017). Chlorinated solvents were added stepwise with initially approximately 200 μM, and subsequently 400 and 600 μM, added after the previous dose was converted. Cultivation in transfers 0, 1, and 2 was conducted using 100 mL mineral salt medium in 240 mL serum bottles (Supplementary Figures S1B,C). Upscaling of cultivation (transfer 1, Supplementary Figure S1D) was done with 500 mL mineral salt media in 1 L serum bottles. All cultivation bottles were flushed with N_2_ and CO_2_ (70/30%), closed with Teflon-lined butyl rubber stoppers and aluminum crimp caps and sterilized for 40 min at 120°C. Triplicate bottles were amended with 500 μM PCE, *cis*-DCE, 1,2-DCA or VC, and with another 1 mM of the respective electron acceptor after the first dose was completely consumed. The bottles were inoculated with 5% (v/v) of transfer 0 grown on PCE, *cis*-DCE, 1,2-DCA or VC, respectively. Hydrogen as electron donor was added at 0.5 bar overpressure. Cultures were incubated in the dark without shaking at 20°C.

For proteomic analyses, 30 mL of the culture were processed for LC-MS/MS analysis after each dose of electron acceptor (first: 0.5 mM, second: 1 mM) was completely dehalogenated. Cultures were then transferred to fresh medium with an inoculum of 10% (v/v) and sampling was repeated (Supplementary Figures S1B,C).

### Isotope Fractionation Experiments and Activity Assays

Isotope fractionation experiments and activity assays were conducted using resting cells. Cells were harvested from 400 mL culture (Supplementary Figure S1D) by anoxic centrifugation at 16°C and 5000 × *g* for 1 h. Half of the supernatant was removed anoxically and the residual fraction was centrifuged again under the conditions stated above. Under anoxic conditions, the supernatant was removed completely, the pellet was resuspended in 1 M potassium acetate buffer pH 6.8 and transferred to a sterile anoxic glass vial crimp closed with Teflon-lined butyl rubber stoppers.

Fractionation experiments and activity assays were conducted anoxically using 200 mM potassium acetate buffer pH 6.8, 1 mM methyl viologen and 0.2 mM titanium-III-citrate in 10 mL glass vials, yielding in total 3.9 mL reaction mixture, and crimp closed with Teflon-lined butyl rubber stoppers. 50 μL of the 100 mM electron acceptor stock solution in ethanol were added and the mixture was shaken for 30 min at 20°C in the dark. Another 2 mM titanium-III-citrate and 1 mL of the resuspended pellet were added per vial to start the dehalogenation reaction. Nine vials were amended per set (Supplementary Figure S1E), with one of the electron acceptors PCE, *cis*-DCE, VC or 1,2-DCA, and one of the four resting cell suspensions (derived from cultures amended with one of the electron acceptors, PCE, *cis*-DCE, VC or 1,2-DCA). Additionally, four vials were kept as abiotic control without addition of cells but 1 mL anoxic desalted and bi-distillated water. Vials were shaken during incubation at 30°C in the dark and dehalogenation progress was determined by analysis of electron acceptor and product concentrations in the culture headspace via GC-FID. Vials were sacrificed at different extents of dehalogenation, described as C_t_/C_0_ (where C_t_ is the residual electron acceptor concentration at the sampling time *t* and C_0_ the initial concentration) ranging between 0.1 and 0.9, by addition of 0.5 mL of acidic sodium sulfate solution (280 g⋅L^–1^, pH 1) as described elsewhere (Franke et al., 2017). Sacrificed samples were analyzed using gas chromatography with isotope ratio mass spectrometry.

### Concentration Analysis by Gas Chromatography-Flame Ionization Detection (GC-FID)

Concentrations of PCE, cDCE, 1,2-DCA, VC and the product ethene were determined by analysis of headspace samples via gas chromatography with FID (Varian Chrompack CP-3800, Middleburg, the Netherlands) equipped with a GS-Q-column (30 m × 0.53 mm, J&W Scientific, Waldbronn, Germany) as described previously (Schmidt et al., 2014).

### Analysis of Carbon Isotope Ratios by Gas Chromatography – Isotope Ratio Mass Spectrometry

Carbon isotope ratios were determined via compound-specific stable isotope analyses using GC-isotope ratio mass spectrometry (IRMS). The organic mixture was separated by gas chromatography and baseline-separated compounds were chemically converted into the analyte gas CO_2_ for ^13^C/^12^C analysis (Elsner et al., 2012). Carbon isotope fractionation was determined in triplicate by injection into the gas chromatograph (Agilent 6890, Palo Alto, CA, United States) using a CP-PoraBOND column (50 m × 0.32 mm, 5 μm inner diameter, J&W Scientific, Germany). One mL of the reaction mixture was transferred to a He-flushed 10 mL glass vial wherefrom 1 mL headspace sample was injected via autosampler (TriPlus RSH, Thermo Scientific, Germany) with a split ratio of 1:5. For chromatographic separation a temperature gradient program was used, starting from 30°C (held for 10 min) followed by a 20°C ⋅ min^–1^ gradient to 250°C (held for 5 min) with 2 mL ⋅ min^–1^ flow and an injector temperature of 280°C (Franke et al., 2017). Carbon isotope ratios were determined at the IRMS (MAT 235, Thermo Scientific, Germany) relative to the laboratory reference gas [CO_2_, calibrated against Vienna Pee Dee Belemnite standard V-PDB, IAEA Vienna, Austria (Coplen et al., 2006)]. The overall analytical uncertainty was <0.5%.

### Analysis of Chlorine Isotope Ratios by Gas Chromatography – Multi Collector-Inductively Coupled Plasma Mass Spectrometry

For chlorine isotope analysis a multi collector-inductively coupled plasma mass spectrometer was coupled to gas chromatography (GC-MC-ICPMS) (Horst et al., 2017). The MC-ICPMS, a Neptune (Thermo Fisher Scientific, Germany), was equipped with a gas chromatograph Trace 1310 (Thermo Scientific, Germany) coupled to FID. Triplicate samples were analyzed by manual injection of 0.05–1 mL headspace with a split ratio of 1:10 with injector kept at 250°C and a carrier gas flow of 2 mL⋅min^–1^. For the analysis, a Zebron ZB-1 capillary column (60 m × 0.32 mm i.d., 1 μm film thickness; Phenomenex Inc.) was utilized isothermal at 100°C for 1,2-DCA, VC and *cis*-DCE and 120°C for PCE analysis. The separated compounds were transferred to the plasma via a Thermo Electron TransferLine (AE2080, Aquitaine Electronique, France) heated to 250°C using an auxiliary helium flow of 5 mL⋅min^–1^. Instrument tuning and preparation was performed daily prior to the measurements (Horst et al., 2017). Chlorine isotope ratios were determined relative to the laboratory standards [methyl chloride, TCE-2, and TCE-6 (Horst et al., 2017; Renpenning et al., 2018)]. The overall analytical uncertainty was <0.5%.

### Evaluation of Isotopic Data

Stable isotope composition was reported in δ-notation (%) relative to an international standard (Coplen et al., 2006; Elsner, 2010; Renpenning et al., 2015). The calculation of carbon and chlorine isotope enrichment factors ε^*X*^ (where *X* represents the analyzed element, i.e., C or Cl, respectively) was accomplished according to the Rayleigh-Equation (1) (Mariotti et al., 1981). The concentrations at different time points (t) of the reaction and the starting concentration (0) are represented as C_t_ and C_0_, respectively, whereas *R* = 1 + δ (Coplen et al., 2006).

(1) $$l\,n\,\left(\frac{R_{t}}{R_{0}}\right)=\left(\varepsilon^{X}\right)\times \left(\frac{C_{t}}{C_{0}}\right)$$

The slope derived from the dual-element plot (m_2D_) equals the Λ-value and approximately conforms to the ratio of the enrichment factors of the compared elements (ε^C^/ε^Cl^) according to Eq. 2.

(2) $$\Lambda =m_{2\,D}\approx \frac{\varepsilon^{C}}{\varepsilon^{C\,l}}$$

Based on the slope of the regression a two-tailed *T*-test was uses to calculate the 95% confidence interval (data evaluation in Microsoft Excel).

### Apparent Kinetic Isotope Effects

The previously calculated bulk enrichment factor ε^*X*^_bulk_ describes the isotopic fractionation of the whole molecule. To determine position-specific kinetic isotope effects intra-molecular competition and non-reacting positions have to be taken into account (Elsner et al., 2005). The AKIE values were determined for PCE, cDCE, 1,2-DCA, and VC according to Eq. (3) (Elsner et al., 2005). The number of carbon or chlorine atoms is described by n, the number of atoms at the reactive position by x, whereas z takes the number of indistinguishable reactive sites into consideration. The following values were used for carbon (*n* = 2 for all cases): PCE: *x* = 1, *z* = 4; *cis*-DCE: *x* = 1, *z* = 2; VC: *x* = 1, *z* = 1; 1,2-DCA: *x* = 2, *z* = 2 (concerted) or *x* = 1, *z* = 2 (stepwise). For chlorine, the following values were used: PCE: *n* = 4, *x* = 1, *z* = 4; *cis*-DCE: *n* = 2, *x* = 1, *z* = 2; VC: *n* = 1, *x* = 1, *z* = 1; 1,2-DCA: *n* = 2 and *x* = 2, *n* = 2 (concerted) or *x* = 1, *z* = 2.

(3) $$A\,K\,I\,E=\frac{1}{1+\left(z\times \left(\frac{n}{x}\right)\times \left(\varepsilon_{b\,u\,l\,k}^{X}\right)\right)}$$

### Cell Harvesting and Sample Preparation for Proteomic Analysis

*Dehalococcoides mccartyi* strain BTF08 cultures cultivated with different halogenated electron acceptors PCE, cDCE, 1,2-DCA or VC, were harvested by centrifugation of 30 mL culture at 10,000 × g for 45 min at 16°C. Cell pellets were washed with 100 mM ammonium bicarbonate buffer pH 7.9, centrifuged at 10,000 × g for 30 min at 16°C and subsequent removal of the supernatant. The cell pellet was re-suspended in 30 μL 50 mM ammonium bicarbonate buffer pH 7.9 and the cells were disrupted by three cycles of freezing, using liquid nitrogen, and thawing at 40°C while shaking at 750 rpm. Proteins were reduced using 50 mM dithiothreitol for 1 h at 30°C (400 rpm) and alkylated by 130 mM 2-iodoacetamide for 1 h at 22°C and 400 rpm prior to trypsin digest overnight through 0.6 μg trypsin (Sequencing Grade Modified Trypsin, Promega) at 37°C at 400 rpm. Trypsin digest was stopped by 1 μL 100% formic acid and followed by centrifugation at 13,000 rpm (Centrifuge 5430, rotor FA-45-24-11, Eppendorf, Germany) for 20 min. The pellet was discarded, the supernatant concentrated to 10 μL by vacuum centrifugation at 20 mbar and stored until further processing at −20°C.

Peptides for LC-MS/MS analysis were desalted before injection using ZipTip-μC18 material (Merck Millipore, Germany). Solvents were evaporated under vacuum and samples re-suspended in 0.1% formic acid prior to LC-MS/MS analysis.

### LC-MS/MS and Proteomic Data Analysis

Proteomic analysis was performed via nano-liquid chromatography-tandem mass spectrometry (nLC-MS/MS) using an Orbitrap Fusion (Thermo Fisher Scientific, Bremen, Germany) coupled with a nano ultra-performance liquid chromatography system (Dionex Ultimate 3000RSLC; Thermo Scientific, Germany) and an Acclaim PepMap 100 C18 LC-Column (Thermo Fisher Scientific) as described previously (Goris et al., 2016). For liquid chromatography 0.1% formic acid (eluent A) and 80% acetonitrile with 0.08% formic acid (eluent B) were used and 3 μL of sample was injected. With a constant flow rate of 300 nL⋅min^–1^ 4% eluent A was hold for 6 min prior to increase to 55% eluent A over 120 min being further increase to 90% within 1 min, hold for 4 min and decreased back to 4% eluent A within 1 min.

Subsequent peptide analysis was conducted using Proteome Discoverer (v2.2, Thermo Fisher Scientific, Germany) with the *D. mccartyi* strain BTF08 genome as search database (UniProt Proteome ID: UP000011727) (Pöritz et al., 2013). The mass spectrometry proteomics data have been deposited to the ProteomeXchange Consortium via the PRIDE [1] partner repository with the dataset identifiers PXD018558 and PXD018595.

### Classification Into RdhA Orthologous Groups

Determination of the orthologous groups to which the identified RdhA proteins belong was done via sequence alignment of the data used by Hug et al. (2013) and the proteome of *D. mccartyi* strain BTF08 (UniProt Proteome ID: UP000011727) (Hug et al., 2013; Pöritz et al., 2013) followed by phylogenetic analysis of this alignment. The alignment and phylogenetic tree (Minimum Evolution Tree with 100 bootstrap repetitions) were performed using Mega X (Kumar et al., 2016).

## Results

### Dehalogenation Activity in Growing Cultures Correlates With Electron Acceptor Change

*Dehalococcoides mccartyi* strain BTF08 was cultivated on PCE, cDCE, VC or 1,2-DCA as electron acceptor after growth for at least four transfers with one constant electron acceptor, either PCE, cDCE, VC or 1,2-DCA (Supplementary Figures S1A–C). *D. mccartyi* strain BTF08 adapted fast, with no obvious delay in dehalogenation compared to controls maintained on the same substrate, when the electron acceptor was switched in most cases (Table 1). Delayed dechlorination to ethene (as indicated by the dehalogenation lag time) was observed when switching from 1,2-DCA, cDCE or VC to PCE. The dechlorination activities (the time to dehalogenate 1.5 mM organohalide, see Table 1) were highest with cDCE and VC regardless of the electron acceptor used for pre-cultivation, whereas those with PCE and 1,2-DCA showed stronger variations. Dechlorination activity, however, was decreased when switching from 1,2-DCA to VC as well as from PCE or VC to 1,2-DCA.

**TABLE 1:** Expression of reductive dehalogenase homologous proteins (RdhA).

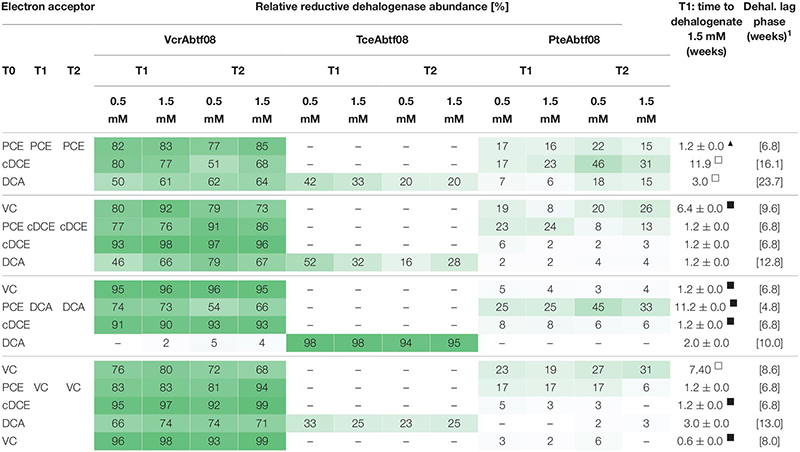

*Effect of changing the electron acceptor from transfer 0 (T0) to transfer 1 (T1) including dehalogenation activity under growth conditions of *D. mccartyi* strain BTF08. The data shows the relative abundance of RdhA proteins normalized against the sum of the abundance of all RdhA proteins after 0.5 mM and in total 1.5 mM dehalogenation of the electron acceptor in transfer 1 (T1) excluding the dehalogenation lag phase [in square brackets]. Lag times were defined by the time at which 25 μM ethene (∼5% of electron accepter dehalogenated) was observed. The intensity of the green shading corresponds to the calculated value. Additionally, time for complete dehalogenation of 1.5 mM electron acceptor in transfer 1 is displayed. ^▲^ One out of three cultures was significantly slower in dehalogenation and therefore excluded; ^■^ one out of three cultures did not dehalogenate and was excluded; ^□^ two out of three cultures did not dehalogenate and were therefore excluded.*

### Analysis of the Expression of RdhA Proteins *in vivo* in Response to Electron Acceptor Change

The expression of RdhA proteins (Table 1) was analyzed in response to the electron acceptor present. Seven RdhA proteins were detected and assigned to orthologous groups (OG) (Supplementary Figure S4). The RdhA_ btf08_185 (NCBI: AGG07294, OG5) and RdhA_btf08_1407 (AGG08472, OG8) correspond to the TCE reductive dehalogenase (TceA_btf08_) and VC reductive dehalogenase (VcrA_btf08_), respectively, previously described (Müller et al., 2004; Pöritz et al., 2013). RdhA_btf08_1393 (AGG08458, OG16) was present in increased abundance if PCE was used as electron acceptor suggesting that it acts as a PCE reductive dehalogenase. As RdhA_btf08_1393 shares 99% amino acid sequence similarity to an identified PteA from *D. mccartyi* strain 11a5 (Zhao et al., 2016) it will be referred to as PteA_btf08_. Thus, RdhA_btf08_1393 (PteA_btf08_) and not the expected RdhA_btf08_1454 (PceA_btf08_, AGG08519), also encoded in the genome (Pöritz et al., 2013), was associated with PCE dechlorination. Minor abundant (less than 2% of the expressed RdhA) and not functionally identified were RdhA_btf08_1481 (AGG08546, OG10), RdhA_btf08_1497 (AGG08562, OG15), RdhA_btf08_1488 (AGG08553, OG17), and RdhA_btf08_121 (AGG07230, OG23).

PteA_btf08_ had a relative high abundance in cultures with PCE as electron acceptor (Table 1, 2). PteA_btf08_ functionality was confirmed as its expression increased if PCE was used as electron acceptor, after transfer of cells pre-grown on cDCE or 1,2-DCA. PteA_btf08_ relative abundance decreased if the electron acceptor was switched from PCE to cDCE or VC (Table 1). Expression of PteA_btf08_ increased once electron acceptor was switched from PCE or VC to 1,2-DCA, although with apparently lower rates for 1,2-DCA dehalogenation (Table 1). VcrA_btf08_ was dominantly expressed in presence of halogenated ethenes but not for the cultures maintained on 1,2-DCA (VcrA_btf08_ ≤ 5%). After transfer from cultures with 1,2-DCA to halogenated ethenes, VcrA_btf08_ expression was increasing with each dose of the respective halogenated ethene. No change in expression was visible after the next transfer anymore. Strikingly, TceA_btf08_ was expressed as almost the only RdhA if 1,2-DCA was the sole electron acceptor during cultivation. The abundance of TceA_btf08_ decreased when the electron acceptor was changed to one of the halogenated ethenes. Interestingly, TceA_btf08_ was not detected in cultures with 1,2-DCA, previously cultivated on the chlorinated ethenes, however, PteA_btf08_ and VcrA_btf08_ were detected.

**TABLE 2:** Substrate specificity of expressed reductive dehalogenases.

Cultivation electron acceptor	Relative abundance of reductive dehalogenases in the culture [%]			Electron acceptor in the resting-cells activity test	Dehalogenation activity
	VcrA_btf08_	TceA_btf08_	PteA_btf08_		
PCE	83	–	17	PCE cDCE 1,2-DCA VC	+++ ++ n.s.d. +++

cDCE	96	–	4	PCE cDCE 1,2-DCA VC	++ ++ + +++

1,2-DCA	2	98	–	PCE cDCE 1,2-DCA VC	+ ++ ++ ++

VC	97	–	3	PCE cDCE 1,2-DCA VC	++ ++ n.s.d. +++

*Activity tests were set up with harvested resting cells from the upscaled transfer 1 cultures (Supplementary Figure S1D). Relative abundance of the dehalogenases are mean value of the two time points in transfer 1 (Table 1). The shown dehalogenation activity represents dehalogenation of 50% of the initial electron acceptor concentration in (+++) less than 5 h, (++) less than 48 h, (+) less than 72 h and with no significant dehalogenation (n.s.d).*

### Substrate Specificity of Differently Induced Cells and Related RdhA Inventory

Subsequently, substrate specificity of the RdhAs was tested using resting cells and the artificial electron donor methyl viologen. Resting cells contained the above described RdhA inventory (Table 2) and were derived from *D. mccartyi* strain BTF08 cultures which were cultivated for several transfers with a single specific electron acceptor (Supplementary Figure S1E). Cells with almost exclusively TceA_btf08_ present, dechlorinated 1,2-DCA, cDCE, and VC effectively and PCE at lower rates completely to ethene (data not shown). 1,2-DCA was dechlorinated in cases when TceA_btf08_ was absent only if cells were cultivated with cDCE, albeit at lower rates (VcrA_btf08_ was present as almost exclusive RdhA). In cells cultivated with PCE or VC as electron acceptor 1,2-DCA was not dehalogenated. All tested halogenated ethenes were dechlorinated in cells when VcrA_btf08_ (>96%) was dominantly present.

### Stable Isotope Fractionation Patterns Suggest Distinct Dehalogenation Mechanisms

**FIGURE 2:**
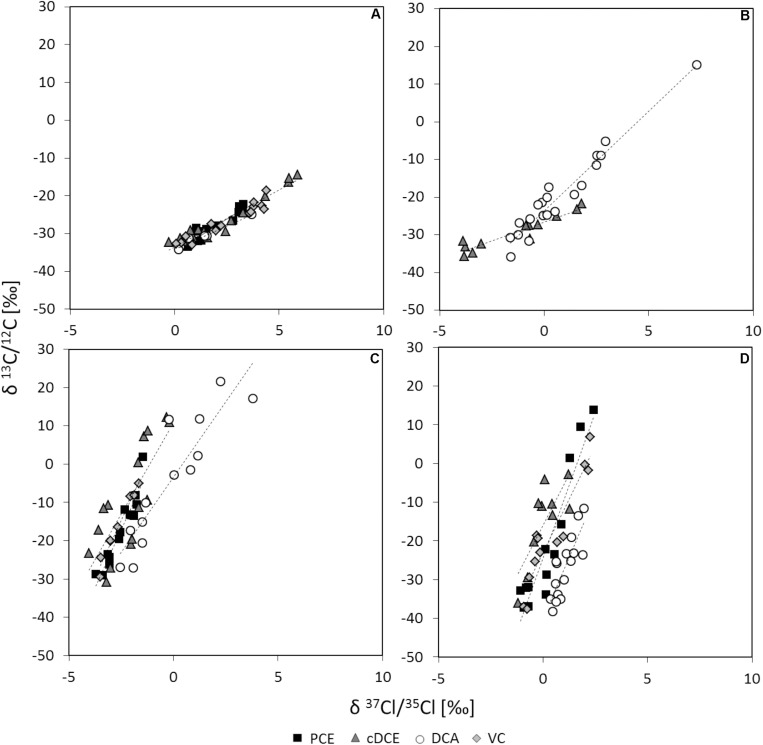
Double-element-plots for stable carbon and chlorine isotope fractionation. Activity assays using resting cells of *D. mccartyi* strain BTF08 with electron acceptors **(A)** PCE, **(B)** 1,2-DCA, **(C)** cDCE or **(D)** VC in dependence of the electron acceptors used for growth in upscaled transfer 1. Linear fits are represented by dashed lines.

The underlying reaction mechanisms for dechlorination of PCE, cDCE, DCA, and VC were characterized by dual carbon and chlorine stable isotope analysis. Stable carbon isotope analysis of cDCE-, DCA- and VC-dechlorination show stronger isotope fractionation compared to PCE-dechlorination (Supplementary Figure S2), independently of the electron acceptor used for cultivation of cells and related RdhA inventory (Table 2). Stable chlorine isotope analysis revealed stronger chlorine isotope fractionation for PCE or DCA compared to cDCE or VC (Supplementary Figure S3). For comparison of the reaction mechanisms, dual-element fractionation patterns were evaluated (Figure 2) and Λ-values, relating the carbon to chlorine isotope fractionation, were calculated. Two main patterns could be observed for halogenated ethenes, (i) relatively strong carbon versus chlorine isotope fractionation for cDCE and VC and (ii) lower carbon vs. chlorine isotope fractionation for PCE. 1,2-DCA dehalogenation showed strong chlorine isotope fractionation either vs. (i) strong carbon isotope fractionation (>98% TceA_btf08_) or vs. (ii) lower carbon isotope fractionation 1,2-DCA (>96% VcrA_btf08_).

Apparent kinetic isotope effects (AKIE) were calculated for the substrate to confirm the difference in reaction of the chlorinated ethenes (Table 3). Relative AKIE^C^/AKIE^Cl^ showed a similar trend with 0.98 to 1.00 for PCE vs. 1.04 to 1.10 for *cis*-DCE and VC. Further, the AKIE for 1,2-DCA was evaluated to investigate whether concerted or stepwise removal of chlorine takes place during dehalogenation (Table 3). For carbon stable isotope analysis, AKIE calculated for concerted 1,2-DCA dechlorination did not differ significantly in comparison to dechlorination of PCE and VC. Chlorine AKIE were similar to those calculated for cDCE and VC but significantly lower than for PCE. Calculated carbon AKIE for stepwise dehalogenation of 1,2-DCA (1.086 to 1.129) were similar to those calculated for cDCE (1.064 to 1.102) and significantly higher than those for PCE (1.015 to 1.042) or VC (1.039 to 1.069). Chlorine AKIE for stepwise 1,2-DCA dehalogenation (1.018 to 1.023) were significantly higher than those for cDCE and VC (1.001 to 1.011) but lower than for PCE dehalogenation (1.030 to 1.061). For stepwise dechlorination of 1,2-DCA all AKIE exceeded the theoretical Streitwieser limit for C-Cl-bond cleavage, KIE^C^ = 1.057 and KIE^Cl^ = 1.013 (Elsner et al., 2005). Stepwise dehalogenation of 1,2-DCA is therefore considered unlikely, agreeing with previous studies (Fletcher et al., 2011; Palau et al., 2017). Overall, the high observed AKIE^C^ for cDCE and VC fit with previous observed isotope fractionation during dechlorination by various *D. mccartyi* strains and *D. mccartyi* containing consortia (Fletcher et al., 2011).

**TABLE 3:** Summary of isotope fractionation analysis.

electron acceptor		carbon			chlorine			2D	
^*L*^1	RC	ε_*C*_ [‰]	*R*^2^	AKIE	ε_*Cl*_ [‰]	*R*^2^	AKIE	Λ	R^2^
PCE	PCE	−5.1 ± 2.1	0.92	1.015 ± 0.012	−2.5 ± 2.6	0.76	1.030 ± 0.014	3.5 ± 0.8	0.88
	1,2-DCA	n.d.			n.d.			n.d.	
	cDCE	−23.3 ± 3.0	0.97	1.102 ± 0.019	−1.9 ± 0.4	0.93	1.006 ± 0.003	12.2 ± 2.5	0.90
	VC	−31.7 ± 3.2	0.98	1.069 ± 0.008	−1.7 ± 0.3	0.98	1.034 ± 0.001	15.1 ± 3.6	0.88
1,2-DCA	PCE	−9.8 ± 1.4	0.97	1.042 ± 0.006	−3.2 ± 0.7	0.89	1.061 ± 0.009	2.5 ± 0.5	0.95
	1,2-DCA	−27.5 ± 4.1	0.92	c: 1.058 ± 0.018 sw: 1.129 ± 0.021	−5.3 ± 0.6	0.96	c: 1.009 ± 0.003 sw: 1.023 ± 0.003	5.3 ± 0.6	0.94
	cDCE	−21.2 ± 2.5	0.96	1.093 ± 0.012	−2.5 ± 1.0	0.75	1.011 ± 0.001	7.9 ± 2.5	0.81
	VC	−18.7 ± 3.4	0.91	1.040 ± 0.008	−1.0 ± 0.5	0.63	1.002 ± 0.001	12.6 ± 4.8	0.69
cDCE	PCE	−8.6 ± 2.0	0.88	1.037 ± 0.009	−1.9 ± 0.4	0.89	1.032 ± 0.008	3.0 ± 0.5	0.94
	1,2-DCA	−18.5 ± 4.2	0.80	c: 1.038 ± 0.022 sw: 1.086 ± 0.024	−4.5 ± 1.6	0.70	c: 1.009 ± 0.003 sw: 1.018 ± 0.005	2.0 ± 0.5	0.89
	cDCE	−24.3 ± 3.1	0.95	1.096 ± 0.030	−1.2 ± 0.4	0.74	1.005 ± 0.002	9.6 ± 4.4	0.63
	VC	−20.0 ± 6.3	0.80	1.039 ± 0.015	−0.4 ± 0.4	0.31	1.001 ± 0.001	10.5 ± 6.8	0.61
VC	PCE	−7.7 ± 1.5	0.90	1.033 ± 0.007	−5.6 ± 1.0	0.94	1.038 ± 0.009	2.7 ± 0.5	0.92
	1,2-DCA	n.d.			n.d.			n.d.	
	cDCE	−14.7 ± 5.1	0.73	1.064 ± 0.024	−1.0 ± 0.5	0.56	1.004 ± 0.002	12.0 ± 2.0	0.97
	VC	−23.7 ± 3.2	0.95	1.051 ± 0.007	−1.6 ± 0.4	0.92	1.003 ± 0.001	11.0 ± 2.9	0.88

*Activity assay using resting cells (RC) with different electron acceptors as for previous six generations comprising enrichment factors for carbon (ε_*C*_), chlorine (ε_*Cl*_) and dual-element Λ-values as well as calculated apparent kinetic isotope effects (AKIE). For 1,2-DCA dehalogenation concerted (c) or stepwise (sw) dehalogenation reactions were taken into consideration. n.d.: not determined as no significant dehalogenation was detected, *R*^2^: correlation coefficient of determination.*

## Discussion

Expression analysis of strain BTF08 under different conditions led to different conclusions of the functional activity of the reductive dehalogenases as assumed previously. For example TceA_btf08_ sharing 96% amino acid sequence identity TceA_195_ (Seshadri et al., 2005; Pöritz et al., 2013), was previously described to be capable of cDCE, VC, TCE, and 1,2-DCA dehalogenation (Magnuson et al., 2000). However, in *D. mccartyi* strain BTF08 TceA_btf08_ dehalogenated 1,2-DCA, cDCE and VC as well as PCE to ethene, albeit at lower rates. Thus, TceA_btf08_ is presumably capable of complete dehalogenation of PCE to ethene. Interestingly, changing the electron acceptor from halogenated ethenes to 1,2-DCA did not lead to expression of TceA_btf08_ although dehalogenation of 1,2-DCA was observed. PCR analysis showed that the gene encoding for TceA_btf08_ was still present in the samples, excluding the possibility that it might have been lost during transfers (data not shown). VcrA_btf08_, highly expressed after 1,2-DCA addition, could have catalyzed the dehalogenation of 1,2-DCA in our experiments, similar to observation for strain VS (Parthasarathy et al., 2015). For dehalogenation of PCE to ethene TceA_btf08_ was not expressed, indicating that it is not needed for this reaction sequence, in contrast to a previous proposal based on genome analysis (Pöritz et al., 2013). PCE was completely dechlorinated to ethene in the presence of PteA_btf08_ and VcrA_btf08_. The tetrachloroethene reductive dehalogenase PteA_btf08_ was expressed in *D. mccartyi* strain BTF08 in the presence of PCE. This was noteworthy, as *pceA* was described in the genome of strain BTF08 (Pöritz et al., 2013) and PceA_btf08_ was thought to catalyze PCE dehalogenation. However, it was not detected within in this study. Consequently, in strain BTF08, PteA_btf08_ is proposed to dechlorinate PCE to TCE, and VcrA_btf08_ presumably dehalogenates TCE, cDCE, and VC to ethene. VcrA from *D. mccartyi* strain VS was described capable of 1,2-DCA (Parthasarathy et al., 2015) and TCE (Müller et al., 2004) dehalogenation, albeit TCE dehalogenation proceeds at significantly lower dehalogenation rates compared to cDCE or VC dehalogenation (Müller et al., 2004).

Based on dual-element CSIA results, the dehalogenation mechanism of the chlorinated ethenes was observed to be dependent on the electron acceptor present in the activity assay. The specific reductive dehalogenases present apparently did not affect mechanism in *D. mccartyi* strain BTF08. This is in contrast to recent findings of Lihl et al. (2019), presenting mechanistic division for TCE dehalogenation if previously cultivated on (i) cDCE or VC (Figure 1Aa) or (ii) TCE or PCE (Figure 1Ab). In this previous study, the presence of *rdhA* genes was analyzed in the investigated consortia, and not for the proteins present. Nevertheless, even when similar *rdhA* genes were dominant, different isotope fractionation patterns were observed (e.g., KB-1 RF and KB-1/VC with dominant *vcrA* but Λ = 2.7 ± 0.2 and 18.2 ± 4.4). Contrarily, dehalogenases without close sequence similarity led to similar dual-element patterns (e.g., KB-1 RF with *vcrA* and Donna II with *tceA* with Λ = 2.7 ± 0.2 and 2.3 ± 0.1, respectively) (Lihl et al., 2019). Thus, investigation of the actual RdhA expressed is essential.

In our study, similar carbon and chlorine isotope fractionation patterns were observed for dechlorination of cDCE and VC, suggesting involvement of a similar reaction mechanism, in accordance with the dechlorination of both, cDCE and VC, by VcrA_btf08_. However, different carbon and chlorine isotope fractionation patterns were observed during dehalogenation of PCE or 1,2-DCA suggesting additional, distinct, dechlorination mechanisms for these two electron acceptors. Studies on the recently isolated PceA from *Sulfurospirillum multivorans* (Bommer et al., 2014) and the VcrA from *D. mccartyi* strain VS (Parthasarathy et al., 2015) proposed long range SET (Figure 1C) whereas inner sphere SET was proposed either by homolytic or heterolytic bond cleavage for NpRdhA from *Nitratireductor pacificus* (Payne et al., 2015) and by homolytic bond cleavage for *D. mccartyi* strain CBDB1 (Cooper et al., 2015; Figure 1Ba,b). Inner sphere nucleophilic substitutions involving covalent cobalt-carbon-intermediates were postulated for halogenated alkenes on the basis of mass spectrometry and isotope fractionation (Heckel et al., 2017, 2018; Figure 1A).

The pronounced carbon isotope fractionation relative to chlorine isotope fractionation for cDCE and VC suggests a nucleophilic substitution-addition reaction via a Co(III)-alkyl-complex (Figure 1Aa). As PCE dehalogenation led to a more pronounced chlorine isotope fractionation relative to carbon isotope fractionation, a nucleophilic substitution-elimination mechanism via a Co(III)-vinyl-complex intermediate was proposed (Figure 1Ab). This difference in reaction between PCE vs. cDCE and VC was also predicted by computational modeling for the reductive dechlorination of chlorinated ethenes with cobalamin (Ji et al., 2017). Heckel et al. (2018) showed that outer sphere SET does not result in chlorine isotope fractionation for halogenated ethenes. Therefore, this mechanism may be excluded for dechlorination of PCE, cDCE and VC in *D. mccartyi* strain BTF08, as we observed significant chlorine isotope fractionation. However, chlorine isotope fractionation was reported previously for PCE dehalogenation in *S. multivorans* (Renpenning et al., 2014) for which long range SET is currently proposed (Kunze et al., 2017). Recent DFT (density function theory) modeling led to the proposition of a concerted chlorine removal and hydrogen insertion from a single [cobalt^…^halogen^…^carbon] transition state, either in inner sphere SET or by long range SET (Johannissen et al., 2017). Contradictorily to the studies by Heckel et al. (2018), the herein determined isotope fractionation pattern may be also explained in dependence of whether the insertion or the abstraction is taking place as rate-limiting step.

1,2-DCA dehalogenation in the presence of solely TceA_btf08_ showed both pronounced chlorine and carbon isotope effects which cannot be explained with a reaction via nucleophilic substitution. 1,2-DCA was previously shown to be dechlorinated via dihaloelimination presumably following a concerted carbon-chlorine bond cleavage (Franke et al., 2017). An outer sphere SET (Figure 1C; Heckel et al., 2017) as well as an inner sphere SET are possible only if two electrons can be transferred almost concurrently and the remaining alkyl-radical can be stabilized sufficiently within the active site of the RdhA. Alternatively, a Co-halogen-bond formation with concomitant removal of vicinal halogens excludes the need for radical stabilization as proposed for NpRdhA (Payne et al., 2015) and is therefore assumed most likely (Figure 1D). Dual-element stable isotope patterns for 1,2-DCA dehalogenation are in close proximity to those determined for PCE dehalogenation, pointing toward an initial C-Cl-bond breakage. In our study, two main patterns are observed corresponding with previous observations for a *Dehalogenimonas*-containing microcosms (Λ = 1.89 ± 0.02) and *Dehalococcoides*-containing cultures (Λ = 6.8 ± 0.2; 6.9 ± 1.2) (Palau et al., 2017; Lihl et al., 2019). The differences in the Λ-values for 1,2-DCA dehalogenation in dependence on the most abundant RdhA can presumably be explained by (i) different underlying dehalogenation mechanisms or (ii) a different stabilization of intermediates within, the most likely different, active site of both RdhA. Latter assumption fits with differences between TceA_btf08_ and VcrA_btf08_, sharing 38% amino acid sequence identity, only (data not shown).

Isotope analyses allowed to characterize the underlying reaction, indicating distinct mechanisms for (i) PCE, (ii) cDCE and VC, and (iii) 1,2-DCA dechlorination. In accordance, our findings indicate that only two reductive dehalogenases, PteA_btf08_ and VcrA_btf08_, are needed for complete PCE dehalogenation to ethene in strain BTF08. TceA_btf08_ was furthermore presumably identified capable of complete PCE dehalogenation to ethene, as well as for 1,2-DCA dehalogenation, although solely expressed if 1,2-DCA was present. Our findings support the previous proposed diversity in reaction mechanism for RdhA and reinforce CSIA as a powerful tool to elucidate biochemical reaction mechanisms. Furthermore, our findings contribute to a pronounced understanding of the underlying dehalogenation processes in anoxic zones, necessary to achieve applicability from laboratory micro-scale experiments to field site remediation approaches.

## Data Availability Statement

The datasets presented in this study can be found in online repositories. The names of the repository/repositories and accession number(s) can be found at: http://www.proteomexchange.org/, PXD018558 and PXD018595.

## Author Contributions

IN and SF developed the overall concept. IN, LA, and SF conceived the experimental study. SF conducted the cultivation, activity tests, isotope fractionation experiments, proteomics sample preparation, analyzed and evaluated the data for activity tests and isotope fractionation, evaluated proteomics data, and developed the first draft. LA and KS conducted phylogenetic analysis. All authors edited and approved the manuscript.

## Conflict of Interest

The authors declare that the research was conducted in the absence of any commercial or financial relationships that could be construed as a potential conflict of interest.
