# Supplementary material for: Dual Element (C/Cl) Isotope Analysis Indicates Distinct Mechanisms of Reductive Dehalogenation of Chlorinated Ethenes and Dichloroethane in Dehalococcoides mccartyi Strain BTF08 With Defined Reductive Dehalogenase Inventories
Source: Front Microbiol. 2020 Jul 17;11:1507. doi: 10.3389/fmicb.2020.01507 (PMC7396605; doi:10.3389/fmicb.2020.01507)
Supplement: Supplementary file 1 [file Data_Sheet_1.PDF]

Supplementary Material to

Dual element (C/Cl) isotope analysis indicates distinct mechanisms of reductive dehalogenation of chlorinated ethenes and dichloroethane in *Dehalococcoides mccartyi* strain BTF08 with defined reductive dehalogenase inventories

Steffi Franke[1], Katja Seidel[1], Lorenz Adrian[1,2], Ivonne Nijenhuis[1*]

[1] Department of Isotope Biogeochemistry, Helmholtz Centre for Environmental Research - UFZ, Permoserstraße 15, 04318 Leipzig, Germany

[2] Chair for Geobiotechnology at TU Berlin

[*]corresponding author: ivonne.nijenhuis@ufz.de

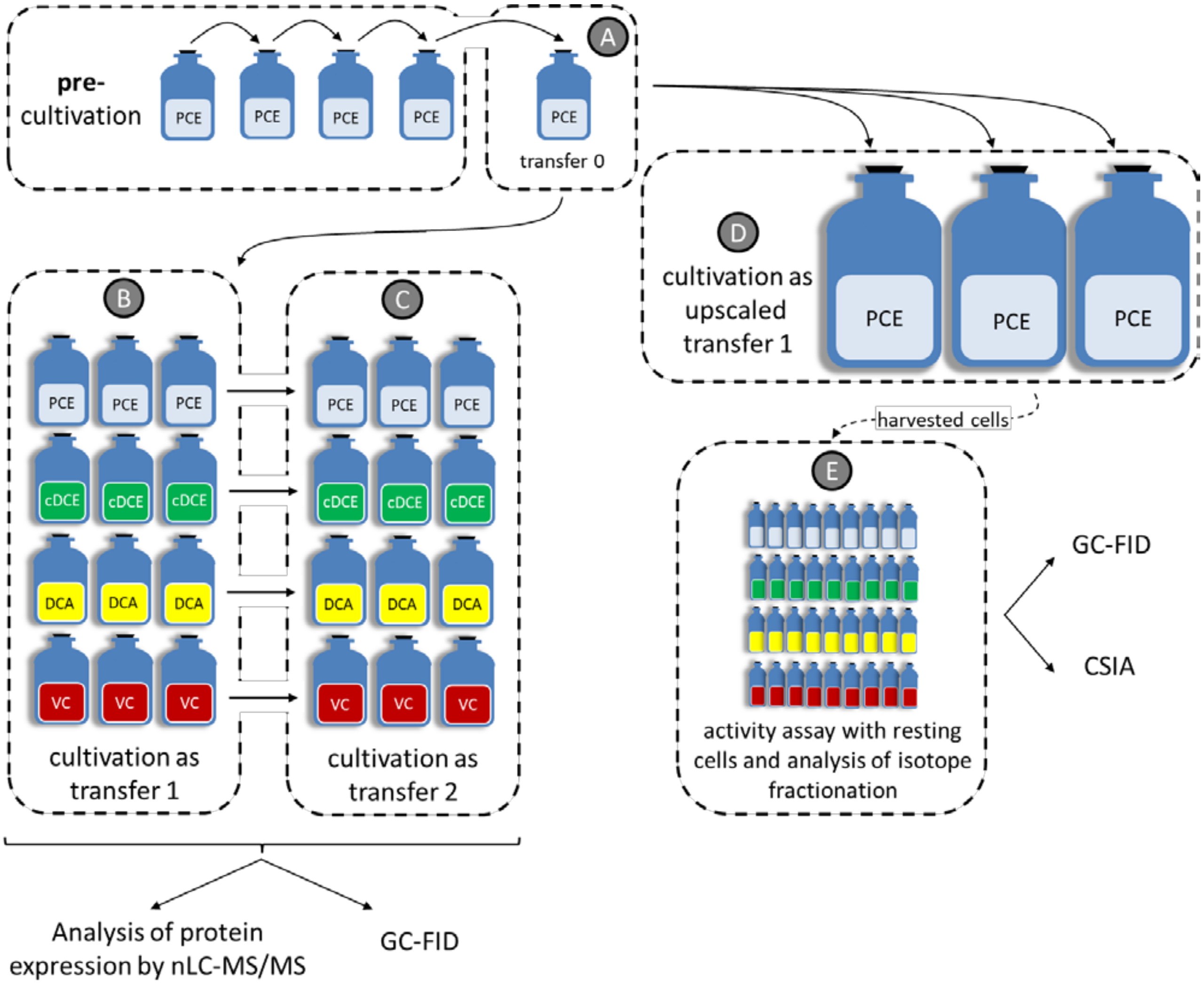

Figure S1: Experimental setup exemplified for pre-cultivation with PCE. Identical procedures were done for pre-cultivation with cDCE, DCA and VC.

D. mccartyi strain BTF08 was cultivated with PCE, cDCE, DCA or VC for at least 4 passages to generate one single starter culture for the four electron acceptor (pre-cultivation, “transfer 0”, A). From these starter cultures we generated cultures for protein expression analysis (B and C) and cell material for activity tests (D and E).

For proteomic analysis the starter cultures were transferred twice in medium with one of the four electron acceptors (transfer 1 and 2, B and C) to monitor the effect of the electron acceptor on RdhA expression. Cells were transferred when the electron acceptor was completely dehalogenated. Proteomic analysis was done from cultures from both transfers (transfer 1 and transfer 2).

For activity test and isotope fractionation analysis, transfer 0 cultures were used to inoculate cultures with larger volumes for all four electron acceptors (shown only for PCE, D). This cell material was used for activity tests (E) which were analyzed after varying incubation times for dehalogenation products and isotopic signature of residual electron acceptors.

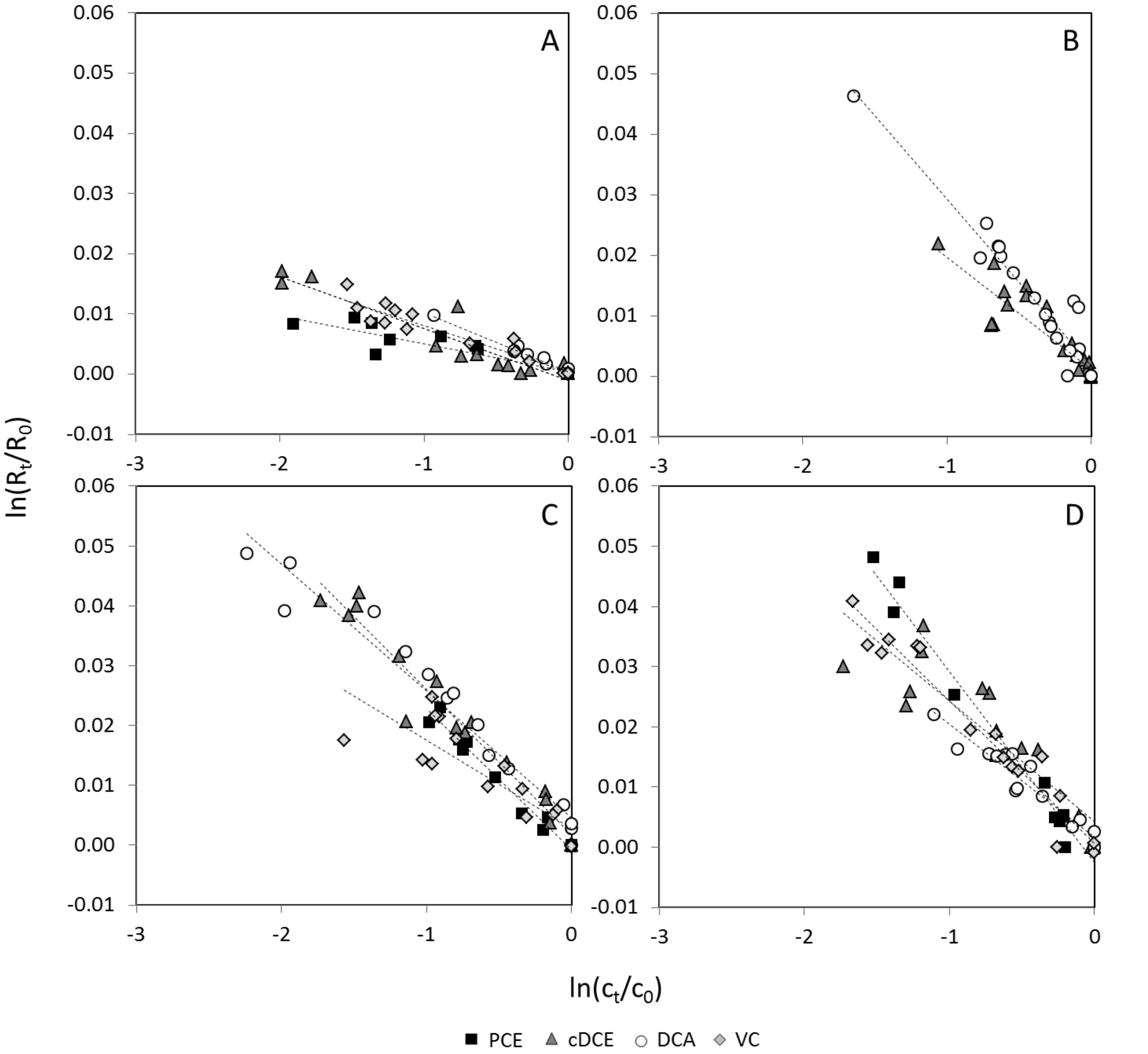

Figure S2: Rayleigh-Plots for stable isotope carbon fractionation. Activity assays using resting cells of *D. mccartyi* strain BTF08 with the electron acceptors (A) PCE, (B) 1,2-DCA, (C) cDCE or (D) VC in dependence of the electron acceptors used for growth in upscaled transfer 1. Linear fits are represented by dashed lines.

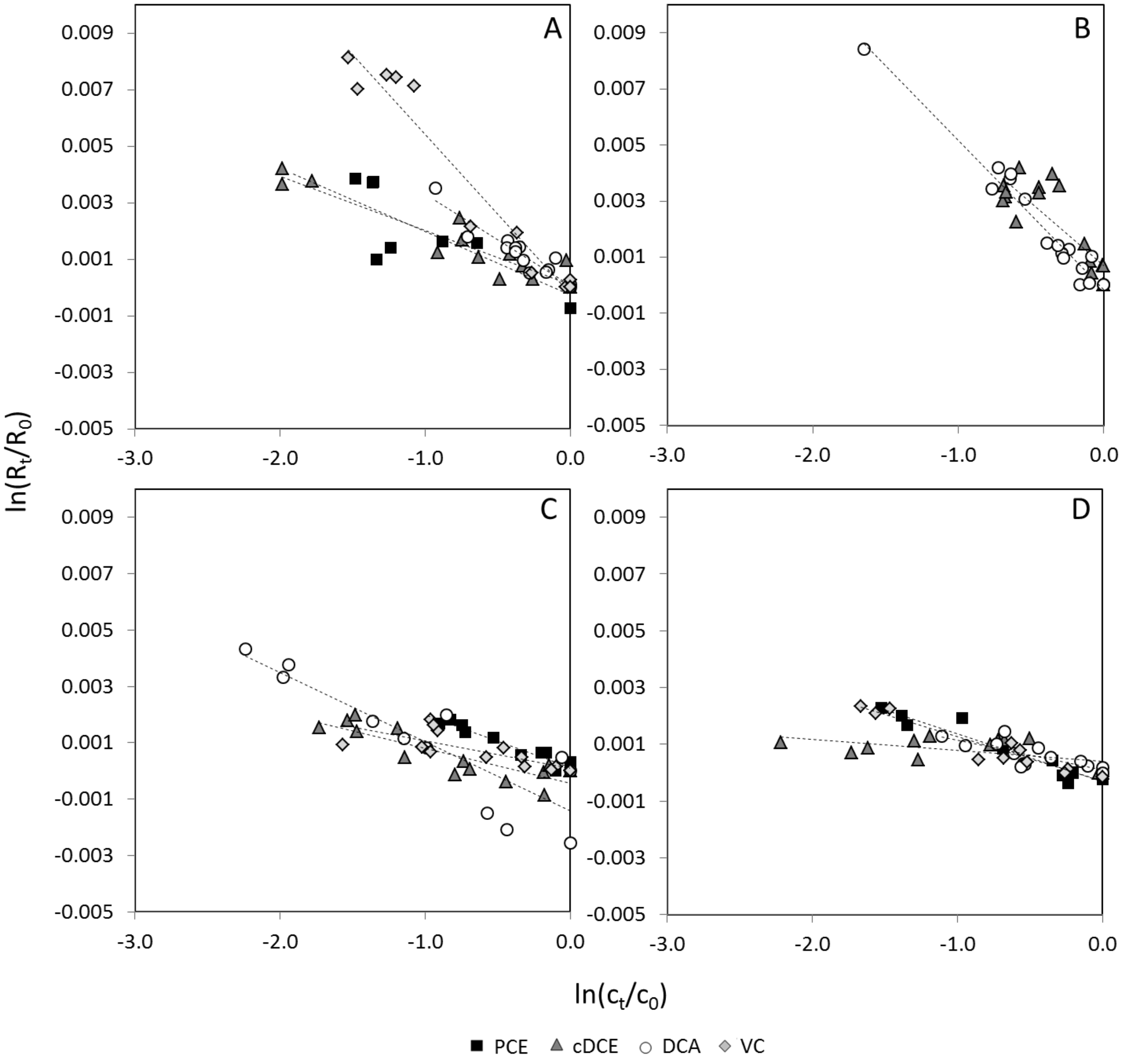

Figure S3: Rayleigh-Plots for stable isotope chlorine fractionation. Activity assays using resting cells of *D. mccartyi* strain BTF08 with the electron acceptors (A) PCE, (B) 1,2-DCA, (C) cDCE or (D) VC in dependence of the electron acceptors used for growth in upscaled transfer 1. Linear fits are represented by dashed lines.

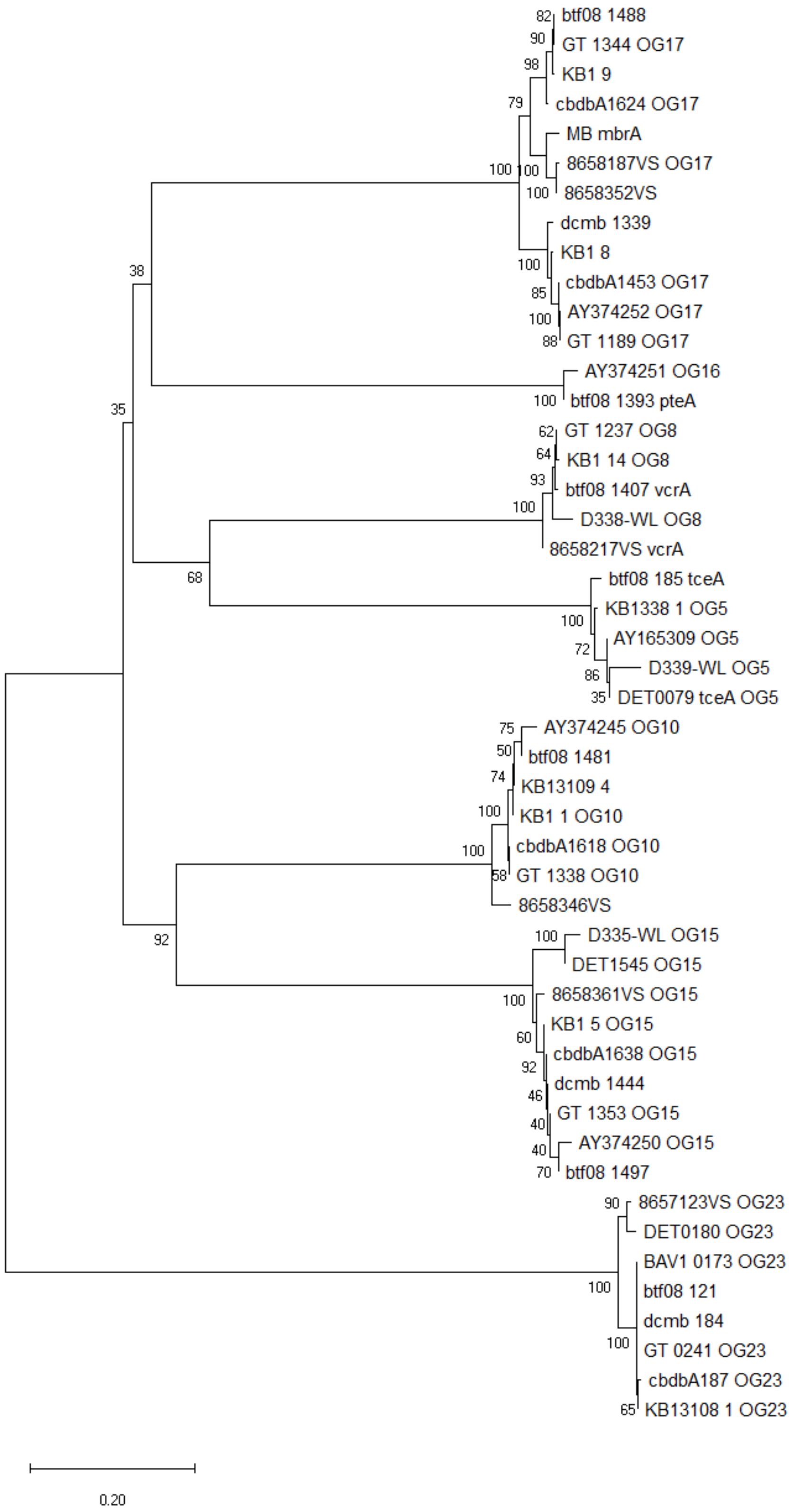

btf08 1488
GT 1344 OG17
KB1 9
cbdbA1624 OG17
MB mbrA
8658187VS OG17
8658352VS
dcmb 1339
KB1 8
cbdbA1453 OG17
AY374252 OG17
GT 1189 OG17
AY374251 OG16
btf08 1393 pteA
GT 1237 OG8
KB1 14 OG8
btf08 1407 vcrA
D338-WL OG8
8658217VS vcrA
btf08 185 tceA
KB1338 1 OG5
AY165309 OG5
D339-WL OG5
DET0079 tceA OG5
AY374245 OG10
btf08 1481
KB13109 4
KB1 1 OG10
cbdbA1618 OG10
GT 1338 OG10
8658346VS
D335-WL OG15
DET1545 OG15
8658361VS OG15
KB1 5 OG15
cbdbA1638 OG15
dcmb 1444
GT 1353 OG15
AY374250 OG15
btf08 1497
8657123VS OG23
DET0180 OG23
BAV1 0173 OG23
btf08 121
dcmb 184
GT 0241 OG23
cbdbA187 OG23
KB13108 1 OG23
0.20

Figure S4: Evolutionary relationships of taxa representing the expressed RdhA proteins from *D. mccartyi* strain BTF08. The evolutionary relationship was determined using the Minimal Evolution method (Rzhetsky and Nei, 1992) computed by MEGA7 (Kumar et al., 2016). The percentage of replicate trees in which the associated taxa are clustered together in the bootstrap test including 500 replicates are shown next to the branches and the tree is drawn to scale, with branch lengths in the same units as those of the evolutionary distances used to infer the phylogenetic tree. Involving 48 amino acid sequences the final dataset comprised 344 positions, gaps and missing data were eliminated.

References

Kumar, S., Stecher, G., and Tamura, K. (2016) MEGA7: Molecular Evolutionary Genetics Analysis Version 7.0 for Bigger Datasets. *Mol Biol Evol* **33**: 1870-1874.

Rzhetsky, A., and Nei, M. (1992) A Simple Method for Estimating and Testing Minimum-Evolution Trees. *Mol Biol Evol* **9**: 945-967.
